# Clinical concept recognition: Evaluation of existing systems on EHRs

**DOI:** 10.3389/frai.2022.1051724

**Published:** 2023-01-13

**Authors:** Juan Antonio Lossio-Ventura, Ran Sun, Sebastien Boussard, Tina Hernandez-Boussard

**Affiliations:** ^1^Biomedical Informatics Research, Stanford University, Stanford, CA, United States; ^2^National Institute of Mental Health, National Institutes of Health, Bethesda, MD, United States; ^3^College of Engineering, Boston University, Boston, MA, United States; ^4^Department of Biomedical Data Sciences, Stanford University, Stanford, CA, United States; ^5^Department of Surgery, Stanford University, Stanford, CA, United States

**Keywords:** clinical concept recognition, electronic health records, natural language processing, clinical information extraction, UMLS, named-entity recognition

## Abstract

**Objective:**

The adoption of electronic health records (EHRs) has produced enormous amounts of data, creating research opportunities in clinical data sciences. Several concept recognition systems have been developed to facilitate clinical information extraction from these data. While studies exist that compare the performance of many concept recognition systems, they are typically developed internally and may be biased due to different internal implementations, parameters used, and limited number of systems included in the evaluations. The goal of this research is to evaluate the performance of existing systems to retrieve relevant clinical concepts from EHRs.

**Methods:**

We investigated six concept recognition systems, including CLAMP, cTAKES, MetaMap, NCBO Annotator, QuickUMLS, and ScispaCy. Clinical concepts extracted included procedures, disorders, medications, and anatomical location. The system performance was evaluated on two datasets: the 2010 i2b2 and the MIMIC-III. Additionally, we assessed the performance of these systems in five challenging situations, including negation, severity, abbreviation, ambiguity, and misspelling.

**Results:**

For clinical concept extraction, CLAMP achieved the best performance on exact and inexact matching, with an F-score of 0.70 and 0.94, respectively, on i2b2; and 0.39 and 0.50, respectively, on MIMIC-III. Across the five challenging situations, ScispaCy excelled in extracting abbreviation information (F-score: 0.86) followed by NCBO Annotator (F-score: 0.79). CLAMP outperformed in extracting severity terms (F-score 0.73) followed by NCBO Annotator (F-score: 0.68). CLAMP outperformed other systems in extracting negated concepts (F-score 0.63).

**Conclusions:**

Several concept recognition systems exist to extract clinical information from unstructured data. This study provides an external evaluation by end-users of six commonly used systems across different extraction tasks. Our findings suggest that CLAMP provides the most comprehensive set of annotations for clinical concept extraction tasks and associated challenges. Comparing standard extraction tasks across systems provides guidance to other clinical researchers when selecting a concept recognition system relevant to their clinical information extraction task.

## 1. Introduction

The ubiquity of electronic health records (EHRs) has created an excessive amount of digital clinical data for research (Evans, 2016). EHRs store structured health information in various formats and unstructured patient data such as progress notes and discharge summaries, account for more than 80% of the data (Murdoch and Detsky, 2013; Assale et al., 2019). These data include critical information about clinical decisions made on patients' diagnosis, prescribed medications, clinical procedures, and its related anatomical locations. Information from these unstructured data is sparse and conversion of these unstructured data to structured data is labor-intensive and expensive (Hersh et al., 2013). Tools have been developed to make use of these data and solve many of the biomedical text mining problems.

Natural language processing (NLP) techniques have been successful in advancing biomedical and clinical research by decreasing the time and effort to obtain critical information from clinical notes (Yim et al., 2016; Wang Y. et al., 2018). Clinical concept recognition, also known as named entity recognition, is a fundamental NLP task that aims to automatically recognize and classify concepts from clinical narratives such as disease diagnosis and medications. Over the past several years, concept recognition for the general domain has attracted considerable attention, and studies applying it to the clinical domain have also emerged (Uzuner et al., 2010, 2011; Pradhan et al., 2014, 2015). Concept recognition systems in clinical settings is crucial because it reduces the manual effort to review patients historical medical record, promotes information exchange across different EHR systems, efficiently summarizes the patient medical history, and help providers as well as patient quickly grasp patient information about their disease conditions, procedures performed, and medications used.

Several concept recognition systems have been proposed to extract clinical information from text to facilitate patient care and clinical research, such as MedLEE (Friedman et al., 1994; Friedman, 2000), MetaMap (Aronson and Lang, 2010), MetaMap Lite (Demner-Fushman et al., 2017), KnowledgeMap (Denny et al., 2003), Apache cTAKES (Savova et al., 2010; Kovačević et al., 2013), HiTEX (Zeng et al., 2006), NCBO Annotator (Jonquet et al., 2009), NOBLE (Tseytlin et al., 2016), ScispaCy (Neumann et al., 2019), MedTagger (Liu et al., 2013), CLAMP (Soysal et al., 2017), QuickUMLS (Soldaini and Goharian, 2016), Doc2Hpo (Liu et al., 2019), medspaCy (Eyre et al., 2021), EHRKit (Li et al., 2022), biomedical and clinical models of Stanza (Zhang et al., 2021), UmlsBERT (Michalopoulos et al., 2021), CancerBERT (Zhou et al., 2022), among others (Doan et al., 2014; Ford et al., 2016; Kreimeyer et al., 2017; Cho et al., 2020). Most of these clinical annotation systems rely on existing health and biomedical vocabularies such as Unified Medical Language System (UMLS) (Bodenreider, 2004) to perform a pattern matching to determine what information to extract and how to encode the extracted information. Documents in the EHRs often contain information that are challenging to extract such as negated sentences, abbreviations and acronym (Kaufman et al., 2016; Assale et al., 2019), and symptoms along with the terms describing their severity (Meystre et al., 2008); extracting such information is important to guide treatment and make informed decisions. For instance, prostate cancer patients treated by surgery, can report mild, moderate or severe urinary incontinence, quantifying this symptom can help determine the best treatment option either use protective pads or surgery (Bozkurt et al., 2020).

Several studies have compared the performance of concept recognition systems; however, they are typically developed internally and may be biased due to different internal implementations, parameters used, and limited number of systems included in the evaluations (Hassanzadeh et al., 2016; Gehrmann et al., 2018; Reátegui and Ratté, 2018; Wang X. et al., 2018). Thus, there is a lack of evidence on these systems' performances used by external scenarios (end-users) and for different clinical concept extraction tasks to support the most appropriate and suitable system for a particular clinical task. This study presents a comprehensive comparison of six concept recognition systems commonly used in the clinical and biomedical domain. We hypothesize that there is not a single system with the best performance over all clinical concept recognition tasks and challenges. We evaluate them using two datasets: the 2010 i2b2/VA challenge dataset for test, treatment, and problem concept extraction (Uzuner et al., 2011), and a sample drawn from the MIMIC-III (Medical Information Mart for Intensive Care III) (Johnson et al., 2016, 2018) clinical care database for problem, treatment, test, and anatomy concept extraction. We also evaluate how these systems handle known extraction challenges. This work fills a gap in the literature providing an external evaluation comparing concept recognition systems at extracting clinical concepts and known challenges on two clinical datasets.

## 2. Materials and methods

**Figure 1:**
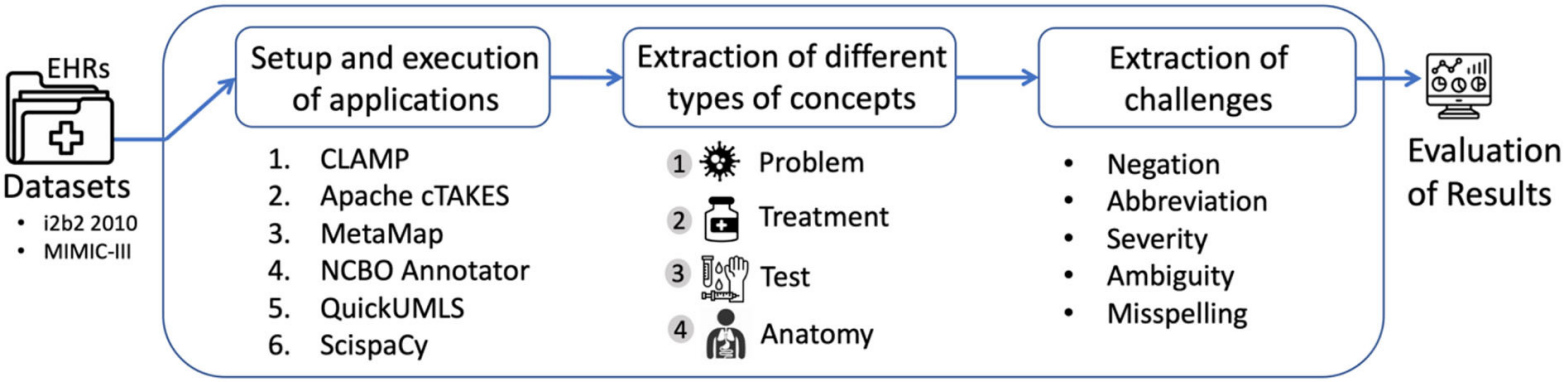
Workflow of the comparison of concept recognition systems across different tasks.

We investigated six biomedical/clinical concept recognition systems: (1) CLAMP, (2) Apache cTAKES, (3) MetaMap, (4) NCBO Annotator, (5) QuickUMLS, and (6) ScispaCy for the extraction of clinical concepts from unstructured EHR data using two datasets: i2b2 and MIMIC-III sample. We evaluated the performance on extracting clinical concepts, including problem (disease or disorder), treatment (procedure and drug), test, and anatomy. Additionally, we used MIMIC-III to examine the performance on how these systems handle known challenges, including abbreviations, negations, severity, ambiguity, and misspellings. Figure 1 outlines the workflow with the basic steps for this evaluation.

### 2.1. Datasets

Table 1 presents the detailed information of the two datasets.

**Table 1:** Details of the i2b2 and MIMIC-III datasets.

	**i2b2**	**MIMIC-III**
Clinical records	Training: 170 Test: 256 Total: 426	Total: 27
Number of concepts	31,161	5,503
Concept types	Problem, treatment, and test	Problem, treatment, test, and anatomy
Total number of tokens	267,249	55,225
Average of number of tokens per note	1,043.9	2,045.4

#### 2.1.1. i2b2

The 2010 i2b2 de-identified annotated dataset is composed of discharge summaries and progress reports from Beth Israel Deaconess Medical Center and University of Pittsburgh Medical Center (Uzuner et al., 2011). i2b2 is publicly-available and is used to evaluate several tasks based on clinical NLP methods such as assertion classification, and relation classification. In our study, we used the test partition of the i2b2 dataset for concept extraction which focused on the extraction of medical concepts such as problems, tests, and treatments from patient reports.

#### 2.1.2. MIMIC-III

We used a sample drawn from MIMIC-III (Johnson et al., 2016, 2018) dataset that included clinical notes of patients in the ICU. We were interested in evaluating the different concept patterns from the health outcomes in patients receiving two chemotherapy agents, docetaxel and cisplatin. Docetaxel-cisplatin combination is a treatment option for specific types of aggressive cancer (Fan et al., 2013). Patients treated with the docetaxel-cisplatin were more likely to experience side effects such as anemia, nausea/vomiting, thrombocytopenia, etc. (Li et al., 2017), thus, their EHRs contained more symptoms and problems. A total of 27 clinical notes were included from MIMIC-III and were manually annotated by a clinical expert for four clinical concepts (Table 1), as well as for abbreviations, negations, severity, ambiguity, and misspellings (Table 2).

**Table 2:** Details and examples of sentences annotated in the MIMIC-III dataset.

	**Number of sentences**	**Number of concepts**	**Example sentences**	**Annotation**
Abbreviation	123	169	He had a CXR that demonstrated possible left base consolidation	CXR: chest x-ray
Negation	169	228	She did not have fevers or chills until the day prior to admission when she noted chills	Fever, chills.
Severity	48	53	At least moderate pulmonary hypertension	Moderate
Ambiguity	26	26	He was then brought to the [^**^Hospital1 18^**^] ED for further management	ED: emergency department
Misspelling	43	43	Metastatic osteogenic sarcoma	Metastatic: metastatic

### 2.2. Concept recognition tools

We set up six clinical concept systems as described below.

#### 2.2.1. CLAMP

CLAMP is a Java-based clinical language annotation, modeling, and processing toolkit (CLAMP, 2021). CLAMP provides NLP modules, such as entity recognition, entity linking, normalization. It presents three different types of concept recognition methods: (1) a deep learning-based model that uses a recurrent neural network (RNN) within the bidirectional LSTM-CRF architecture; (2) a dictionary-based approach with comprehensive lexicon such as the UMLS; and (3) a regular expression-based algorithm to extract concept with common patterns. CLAMP includes NegEx (Chapman et al., 2001), a regular expression algorithm to identify negations. Moreover, additional negation lexicons and rules can be added.

#### 2.2.2. cTAKES

Clinical Text Analysis and Knowledge Extraction System (cTAKES) is an open-source NLP system that combines rule-based and machine learning techniques to extract clinical information from EHR unstructured text (Savova et al., 2010; Kovačević et al., 2013). cTAKES executes some components in sequence to process clinical texts and mainly uses SNOMED-CT (Apache cTAKESTM, 2021). cTAKES also offers the extraction of negated concepts integrating NegEx.

#### 2.2.3. MetaMap

MetaMap is a program providing access to the concepts in the unified medical language system (UMLS) Metathesaurus from biomedical text. It provides a link between the text of biomedical literature and the knowledge, including synonymy relationships, embedded in the Metathesaurus (Aronson and Lang, 2010; MetaMap, 2021). MetaMap includes the NegEx algorithm to extract negated concepts and allows the addition of new rules to identify negations.

#### 2.2.4. NCBO annotator

The National Center for Biomedical Ontology (NCBO) Annotator is a publicly available Web service to process biomedical text and identify ontology concepts from over 1,100 ontologies (Jonquet et al., 2009; NCBO Annotator, 2021). The annotation is based on a syntactic concept recognition tool which uses concept names and synonyms. Moreover, new annotation features are provided through NCBO Annotator+, such as annotation scoring, negation detection (with NegEx/ConText algorithm), and temporality recognition (Tchechmedjiev et al., 2018).

#### 2.2.5. ScispaCy

ScispaCy is a specialized Python NLP library for processing biomedical, scientific, and clinical texts (ScispaCy, 2021) which leverages the spaCy library (spaCy, 2021). ScispaCy is based on word embeddings and deep learning that uses a convolutional neural network (CNN) architecture. It contains three core released packages trained on biomedical text: (1) “en_core_sci_sm” with 100 k terms approximatively as vocabulary and no word vectors; (2) “en_core_sci_md” with 360 k terms as vocabulary and 50 k word vectors; and (3) “en_core_sci_lg” with 785 k terms approximatively as vocabulary and 600 k word vectors (Neumann et al., 2019; spaCy, 2021). ScispaCy does not include negation extraction and matches only 3-g terms to UMLS.

#### 2.2.6. QuickUMLS

QuickUMLS is an unsupervised method for biomedical concept extraction (Soldaini and Goharian, 2016; QuickUMLS, 2021). QuickUMLS uses a simple and efficient algorithm for approximate dictionary matching designed for similarity measures such as cosine, Dice, Jaccard, and overlap coefficients (Okazaki and Tsujii, 2010). QuickUMLS does not provide the functionality to extract negations and uses a subset of over 6 million concepts from UMLS.

### 2.3. Evaluation of clinical concept recognition systems

Using the two datasets, we evaluated the performance of the six concept recognition systems on extracting concepts, including problem, treatment, test, and anatomy. Additionally, we used MIMIC-III to examine the performance of the six systems in five challenging situations, including abbreviations, negations, severity, ambiguity, and misspellings. Results were compared among systems capable of addressing corresponding concepts. Sentences that contained the five challenges were annotated by the six clinical concept recognition systems, and the results were compared with the gold standard.

Evaluation of performance used the exact and inexact match of concepts (Uzuner et al., 2011). Exact means we only consider it correct when phrase boundaries and concept names matched exactly. Inexact matching represents a match over the surface string. The micro-averaged precision, recall, and F-score were compared across all systems for all types of concepts in the two datasets. Of note, we performed evaluations with different parameters and options that systems provide.

## 3. Results

### 3.1. Extraction of four clinical concepts

Table 3 shows the performance of the six systems on the two datasets for exact matching. Of note, CLAMP based on deep leaning outperformed the dictionary-based and regular expression-based methods, as well as ScispaCy with “en_core_sci_sm” outperformed the “en_core_sci_md” and “en_core_sci_lg” models. Thus, the following tables show the results of CLAMP based on deep leaning and ScispaCy with “en_core_sci_sm”. By dataset, the performance varied across different systems in the MIMIC-III dataset with an F-score range between 0.03 and 0.39. While in the i2b2 dataset, we observed similar F-scores, ranging between 0.06 and 0.33 except for CLAMP (F-score 0.70). By clinical recognition systems, CLAMP achieved the best performance in both datasets with an F-score of 0.70 and 0.39 followed by ScispaCy with an F-score of 0.33 and 0.29.

**Table 3:** Clinical concept recognition system performance on **exact match** at extracting clinical concepts from the i2b2 and MIMIC-III datasets.

	**i2b2**			**MIMIC-III**		
	**Precision**	**Recall**	***F*** **-score**	**Precision**	**Recall**	***F*** **-score**
CLAMP	**0.73**	**0.68**	**0.70**	**0.28**	**0.67**	**0.39**
cTAKES	0.16	0.24	0.19	0.18	0.37	0.24
MetaMap	0.13	0.46	0.20	0.12	0.58	0.20
NCBO Annotator	0.23	0.11	0.13	0.25	0.14	0.18
QuickUMLS	0.05	0.09	0.06	0.02	0.05	0.03
ScispaCy	0.25	0.54	0.33	0.19	0.64	0.29

Table 4 shows the performance of the systems for inexact matching. In general, all concept extraction systems performed better in inexact matching than exact matching evaluation. By dataset, the performance varied across different systems in the MIMIC-III dataset with an F-score range between 0.07 and 0.50. While in the i2b2 dataset, we observed similar F-scores, ranging between 0.08 and 0.56 except for CLAMP (F-score 0.94). By clinical recognition systems, CLAMP also obtained the best performance in both datasets with an F-score of 0.94 and 0.50 followed by ScispaCy with an F-score of 0.56 and 0.39.

**Table 4:** Clinical concept recognition system performance on **inexact match** at extracting clinical concepts from the i2b2 and MIMIC-III datasets.

	**i2b2**			**MIMIC-III**		
	**Precision**	**Recall**	***F*** **-score**	**Precision**	**Recall**	***F*** **-score**
CLAMP	**0.98**	**0.92**	**0.94**	**0.36**	**0.87**	**0.50**
cTAKES	0.30	0.45	0.35	0.25	0.51	0.34
MetaMap	0.24	0.83	0.36	0.17	0.81	0.28
NCBO Annotator	0.42	0.21	0.25	**0.36**	0.20	0.25
QuickUMLS	0.06	0.18	0.08	0.05	0.12	0.07
ScispaCy	0.41	0.91	0.56	0.26	**0.87**	0.39

### 3.2. Extraction of five challenges

We executed the six systems on the sentences that contained negations, abbreviations, severity, ambiguity, and misspellings in the MIMIC-III dataset and compared to the manually annotated results. Table 5 presents the six systems' performance on exact match at extracting the five challenging situations. We used exact matching since most of the entities are composed of a single word. Overall, there is no single system excelled in all tasks. Instead, each system performed differently in particular tasks. By clinical task, ScispaCy performed best in extracting abbreviation information with an F-score of 0.86, followed by NCBO annotator (F-score: 0.79).

**Table 5:** Clinical concept recognition system performance on **exact match** at extracting five challenges from MIMIC-III.

	**Abbreviation**			**Negation**			**Severity**			**Ambiguity**			**Misspelling**		
	***R***	***P***	***F***	***R***	***P***	***F***	***R***	***P***	***F***	***R***	***P***	***F***	***R***	***P***	***F***
CLAMP	0.61	0.55	0.57	**0.61**	**0.66**	**0.63**	**0.73**	0.73	**0.73**						
cTAKES	0.62	0.53	0.56	0.41	0.49	0.43	0.39	0.56	0.44	0.04	0.04	0.04	0.02	0.02	0.02
MetaMap	0.66	0.59	0.61	0.52	0.52	0.51	0.47	0.62	0.52	**0.16**	**0.16**	**0.16**	0.02	0.02	0.02
NCBO Annotator	0.83	0.78	0.79	0.15	0.32	0.18	0.61	**0.85**	0.68	0.04	0.04	0.04	0.02	0.02	0.02
QuickUMLS	0.38	0.31	0.33				0.38	0.38	0.38	0.00	0.00	0.00	0.24	0.24	0.24
ScispaCy	**0.87**	**0.86**	**0.86**				0.33	0.33	0.33	0.04	0.04	0.04	**0.41**	**0.41**	**0.41**

**Figure 2:**
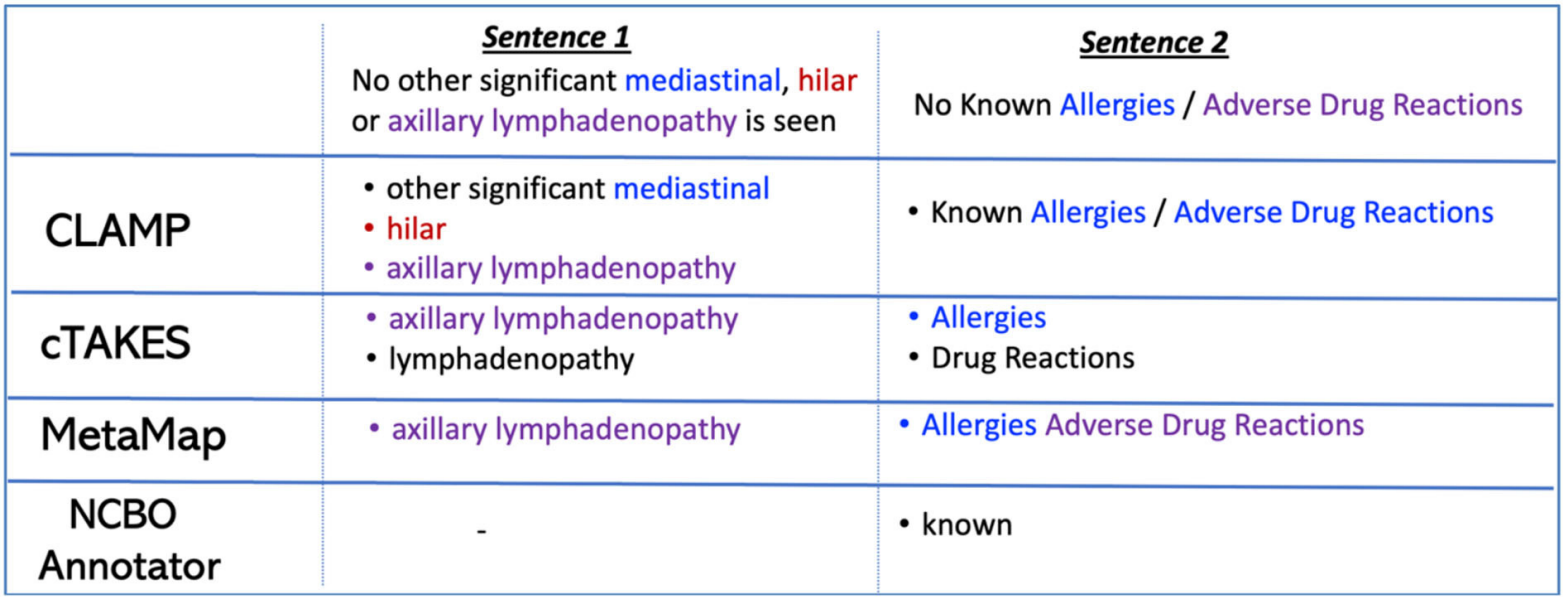
Example of negated concept extraction with four concept recognition systems.

In terms of extracting severity terms and negated concepts, CLAMP achieved the best performance with an F-score of 0.73 and 0.63, respectively. Figure 2 provides two sentences as examples to illustrate how different concept recognition systems extract negated concepts.

When evaluating severity terms, we observed that CLAMP outperformed the other systems. CLAMP was able to identify and categorize severity terms, while the other systems, such as MetaMap, usually identify such terms and categorize them into an UMLS semantic type named “qualitative measure”, which is associated with “T080” as sematic type code (TUI). QuickUMLS identified and assigned these severity modifiers to another semantic type such as “finding” and “intellectual product” associated with TUIs “T033” and “T170”. For example, in the sentence “moderate to severe tricuspid regurgitation”, QuickUMLS identified “moderate” and assigned it “finding” and “intellectual product” as semantic types.

All systems obtained low F-scores for the extraction of ambiguity and misspelling. The list of concepts was composed of ambiguous abbreviations, i.e., abbreviations that were linked to more than one concept. Therefore, the systems were evaluated in terms of the extraction of the abbreviation and their full expansion. The best system for ambiguity abbreviations was MetaMap, which provided the CUI and the expansion of the abbreviation. While CLAMP was able to extract abbreviations, it did not provide the text expansion, only the CUI, therefore, its performance could not be evaluated. ScispaCy performed best at the identification of misspellings and the assignment to the correctly spelled terms. For instance, from the sentence “dilated and severely hypokinetic right ventricle”, the systems are evaluated if they identify the incorrect word “severerly” and the assignment to the correct spelled word “severely”.

## 4. Discussion

Clinical concept recognition is a common NLP task used to extract important concepts from clinical narrative text. There are many systems available to perform this task, yet limited external evaluation exists to guide end-users' selection. This study provides external evaluation of different clinical concept recognition tasks among six well-known systems, including CLAMP, cTAKES, MetaMap, NCBO Annotator, QuickUMLS, and ScispaCy. Our results indicate that CLAMP followed by ScispaCy outperformed the other systems when extracting clinical concepts from clinical notes. Similarly, CLAMP outperformed other systems regarding challenging concept recognition tasks, such as negation and ambiguous abbreviations. We observed that both CLAMP and ScispaCy systems integrate deep learning models (e.g., RNN and CNN architectures, respectively) that were trained using biomedical text, and this may explain the better performance of the systems. Moreover, NCBO Annotator, based on rule-based method, was the second best in clinical concept extraction on the MIMIC dataset as well as in abbreviation and severity recognition.

In our study, the concept recognition systems show better performance in the i2b2 compared to the MIMIC-III dataset. This is likely due to most of the systems used the i2b2 for their training processes and the different annotation process of clinical concepts on the two datasets. For instance, there are concepts composed of determiners in the i2b2 dataset. Determiners refer to those definite or indefinite articles (e.g., “the drop in hematocrit”), possessive pronouns (e.g., “her home medications”), and determinants (e.g., “a broken arm”) before the concept name. The manually annotated MIMIC-III dataset did not include these determiners. Therefore, this indicates that system performance is directly related to the dataset's annotation process.

The systems evaluated in this paper performed differently on handling common challenges, including negated sentences, ambiguous terms, severity descriptions, acronyms/abbreviations, and misspellings. Challenges exist that limits the system performance on certain tasks such as extraction of negated concepts. Some terminologies already included negated concepts as part of their terminology. For instance, the negated concept “no thrombus” exists in SNOMED-CT. Systems that work with up-to-date terminologies first extract concepts that exist already as negated concepts in a terminology, and then they identify when a concept was negated (e.g., no presence of thrombus). In addition, some sentences contain more than one negated concept, and we found systems often fail to extract all negated concepts. If systems were able to extract the set of concept terms, they were often extracted and merged into one single term (see Figure 2, CLAMP extraction on Sentence 2). These issues compromised the system performance, thus a postprocessing step is recommended after concept extraction for an appropriate evaluation of the system performance. Also, CLAMP achieved the best performance in identifying and categorizing severity terms, while other systems, such as MetaMap and QuickUMLS, usually identify these terms and categorize them into a different UMLS semantic type. Thus, a careful evaluation of results of certain systems might be necessary to consider additional semantic types for a complete and correct extraction of severity terms.

All the concept recognition systems we evaluated showed relatively good results at identifying abbreviations. Most of the sentences in our study contained abbreviations that were linked to only one extension, such as “BRBPR” is associated with the extension “bright red blood per rectum”, or “CXR” is associated with “chest x-ray”. Still, we note that ScispaCy and NCBO annotator were more performant in extracting abbreviations than the other systems. ScispaCy depends on deep learning models with its own vocabularies trained on biomedical text, and NCBO annotator extracts more abbreviations since it uses more public terminologies than UMLS (Lossio-Ventura et al., 2019). The extraction of ambiguous abbreviations represents a harder challenge and all applications failed to extract such information, similar to a previous study (Wu et al., 2012). Our ambiguous terms list was composed of ambiguous abbreviations/acronyms, that can be associated with multiple meanings—which is common in clinical and biomedical text (Liu et al., 2015; Lossio-Ventura et al., 2018). For instance, in the sentence “He was then brought to the [^**^Hospital1 18^**^] ED for further management”, all systems correctly identified “ED” as term, however, assigned the expansion/concept “erectile dysfunction” instead of “emergency department”. In addition, many systems rely on the terminology from ULMS, which may not include all clinical abbreviations. Thus, future work in improving extraction of clinical ambiguous abbreviations is needed to ensure the correct interpretation of patient information from clinical notes.

Moreover, there are five important challenges related to concept recognition from clinical text, including negation, severity, abbreviation, ambiguity, and misspellings. These tasks are important for clinical research, and particularly for electronic phenotyping and cohort selection (Banda et al., 2018; Hanauer et al., 2020). Eligibility criteria in clinical cohort may include patients that: did not have an arrhythmia, were diagnosed with coronary artery disease, and are taking some statin. The incorrect identification may lead to an incorrect patient cohort that include patients with the wrong eligibility or exclusion criteria. Such phenotyping is becoming critical, as federal initiative, such as the 21^st^ Century Cures Act, are demanding the use of EHR text data to augment randomized control trials for clinical assertions (Hernandez-Boussard et al., 2019). Therefore, it may be reasonable that each system may be the most appropriate for different research tasks based on its performance.

On the other hand, in overall results were slightly different on both datasets i2b2 and MIMIC-III for exact and inexact match at extracting clinical concepts. As part of future work, other EHR-related datasets should be collected and annotated to allow the performance comparison of diverse clinical concept recognition systems on different datasets. In addition, a common preprocessing task could be added for all the datasets to reduce the noise and improve the recognition of concepts. Finally, named entity recognition tools recently proposed based on new deep learning techniques, such as medspaCy (Eyre et al., 2021), EHRKit (Li et al., 2022), biomedical and clinical models of Stanza (Zhang et al., 2021), and UmlsBERT (Michalopoulos et al., 2021), might be also added for comparison.

## 5. Conclusion

In conclusion, we found that each clinical concept recognition systems perform differently across various clinical tasks. Common challenges exist for all clinical concept recognition systems at extracting ambiguity and misspelling terms. Our work provides a benchmark for different clinical concept extraction systems in an external scenario by end-users that may be useful to other researchers when selecting a concept recognition system relevant to their clinical information extraction task. Our study suggests that CLAMP followed by ScispaCy are more consistent at extracting clinical information on clinical notes related to cancer patients receiving chemotherapy treatment. However, many challenges continue to underscore the performance of such systems, such as medical ambiguity and severity terms.

## Data availability statement

The MIMIC-III dataset is freely available at https://mimic.physionet.org/ whose acquisition involves a required training, data use agreement, and corresponding credentials. The i2b2 (now n2c2) dataset is deidentified and freely available at https://portal.dbmi.hms.harvard.edu/projects/n2c2-nlp/ upon completion of a data user agreement.

## Author contributions

JALV contributed to conceiving the study idea and design, collected the data, set up the applications, and performed the evaluation. SB contributed to configure and evaluate the applications. RS led and performed two rounds of annotation on the MIMIC-III dataset. RS and THB contributed to the study design and provided significant feedback. JALV, RS, and THB wrote the initial draft and revised subsequent versions. All authors read, revised, and approved the final manuscript.
